# Investigation of proteins important for microcirculation using in vivo microdialysis after glucose provocation: a proteomic study

**DOI:** 10.1038/s41598-021-98672-8

**Published:** 2021-09-27

**Authors:** Alexandra Högstedt, Simon Farnebo, Erik Tesselaar, Bijar Ghafouri

**Affiliations:** 1 Department of Surgery in Linköping, and Department of Biomedical and Clinical Sciences, Faculty of Health Sciences, Linköping University, 58185 Linköping, Sweden; 2 Department of Hand Surgery, Plastic Surgery and Burns, and Department of Biomedical and Clinical Sciences, Linköping University, Linköping, Sweden; 3 Department of Medical Radiation Physics, and Department of Health, Medicine and Caring Sciences, Linköping University, Linköping, Sweden; 4 Pain and Rehabilitation Centre, and Department of Health, Medicine and Caring Sciences, Linköping University, Linköping, Sweden

**Keywords:** Proteomics, Preclinical research, Liquid chromatography, Bioinformatics

## Abstract

Insulin has metabolic and vascular effects in the human body. What mechanisms that orchestrate the effects in the microcirculation, and how the responds differ in different tissues, is however not fully understood. It is therefore of interest to search for markers in microdialysate that may be related to the microcirculation. This study aims to identify proteins related to microvascular changes in different tissue compartments after glucose provocation using in vivo microdialysis. Microdialysis was conducted in three different tissue compartments (intracutaneous, subcutaneous and intravenous) from healthy subjects. Microdialysate was collected during three time periods; recovery after catheter insertion, baseline and glucose provocation, and analyzed using proteomics. Altogether, 126 proteins were detected. Multivariate data analysis showed that the differences in protein expression levels during the three time periods, including comparison before and after glucose provocation, were most pronounced in the intracutaneous and subcutaneous compartments. Four proteins with vascular effects were identified (angiotensinogen, kininogen-1, alpha-2-HS-glycoprotein and hemoglobin subunit beta), all upregulated after glucose provocation compared to baseline in all three compartments. Glucose provocation is known to cause insulin-induced vasodilation through the nitric oxide pathway, and this study indicates that this is facilitated through the interactions of the RAS (angiotensinogen) and kallikrein-kinin (kininogen-1) systems.

## Introduction

The interest in studying the vascular effects of the hormone insulin has increased over the last decades. It has been shown that the vascular actions of insulin, to a large extent, are mediated through regulation of endothelium-derived factors^1^, primarily through nitric oxide (NO)-dependent vasodilation and endothelin 1 (ET-1)-dependent vasoconstriction^2–6^. The skin has become a popular model for studying the microvascular function, because it is easily accessible and considered to be a representative vascular bed for the microcirculation^7^. Assessment of the skin microvascular function can be performed by both invasive, for instance microdialysis technique^8,9^, and noninvasive techniques, such as laser speckle contrast imaging (LSCI) or laser Doppler flowmetry^10–12^.

Microdialysis is a well-established technique for measuring local metabolic changes in a target tissue. The basic principle of this technique is to mimic the function of a capillary blood vessel by perfusing a thin dialysis catheter implanted into the tissue with a physiological saline solution. Substances can pass, by simple diffusion, across the dialysis membrane along a concentration gradient^13^. The metabolic and vascular effects of insulin in the human skin have previously been investigated using microdialysis technique^8,9^. It is known that oral glucose provocation, and thereby endogenously increased insulin, increases local tissue blood flow in the human skin. This is partially to increase access to glucose, through a process primarily believed to be mediated by endothelial insulin dependent vasodilation. Measuring the concentration of insulin in the human skin using microdialysis has however appeared to be challenging, because the concentration of insulin in peripheral tissues (intracutaneous and subcutaneous) is low, probably related to decreasing tissue vascularity and blood flow^14^. Altogether, it is known that glucose provocation has microcirculatory effects, partly dependent on the vascular effects of insulin, but the effects on changes in local protein expression are lacking. In order to understand what mechanisms that orchestrate the effects in the microcirculation, investigation of the microdialysate in the search for other markers important for the microcirculation during a glucose provocation is of interest.

The combination of microdialysis and proteomics using mass spectrometry has previously been described, but the number of human studies is limited. The human cutaneous proteome has previously been investigated using large pore microdialysis in two studies^15,16^. Sampling during the trauma phase (caused by the catheter insertion) and during induction of vasodilation and plasma extravasation (by local histamin provocation) resulted in identification of over a hundred proteins, mostly immunoglobulins and different plasma proteins such as apolipoproteins, albumins, complement factors and acute phase proteins^15,16^. This demonstrates that microdialysis combined with shotgun proteomics is a feasible method for studying the complex molecular interactions in the skin. In the search for a therapeutic target for diabetes, the proteome of the subcutaneous interstitial fluid has been investigated using microdialysis and proteomics, identifying elevated galectin-1 in the subcutaneous adipose tissue of type 2 diabetes patients compared to healthy controls^17^. To our knowledge, proteomic on microdialysate from the intravenous compartments has not been investigated before.

By examining the protein expression in microdialysis samples, this study aims to identify protein biomarkers that are related to microvascular changes in different tissue compartments during an oral glucose provocation.

## Material and methods

### Subjects

Eight subjects (4 female), with a mean age of 25 ± 2 years, participated in the study. Subjects were consecutively recruited through advertising on social media. All subjects were healthy, non-smokers and did not use any regular medication, except for oral contraceptives. Detailed numeric data can be found in the supplementary files. The subjects arrived in the morning after an overnight fast and gave their written consent before participation. During the whole experiment, the subjects were reclined in a comfortable position and were only allowed to drink water.

The study was carried out according to the Helsinki declaration and was approved by the regional ethical review board of Linköping (application ID: DNR 2011/362-31 and DNR 2016/122-32).

### Microdialysis

Microdialysis catheters (100 kDa CMA 71 and 20 kDa CMA 67, 10 mm membrane, M dialysis AB, Stockholm, Sweden) were inserted intracutaneous in the volar skin of the non-dominant forearm, subcutaneous in the periumbilical adipose tissue and intravenous in a peripheral vein in each of the eight subjects. The procedure for insertion of microdialysis catheters has previously been described in detail^8,9,14^. Before insertion, all catheters were connected to microinjection pumps (CMA 107, CMA AB, Uppsala, Sweden) and perfused for 90 min to ensure adequate function and reduce the risk of obstructing bubbles in the microdialysis tubing. The perfusate was a control solution consisting of Ringer’s acetate solution with addition of 2.5% albumin and 30 mmol/L urea (APL AB, Umeå Sweden). In the intravenous catheter, 25 IE/mL heparin (Heparin LEO, Malmö, Sweden) was added to the control solution to inhibit clotting, according to the manufacturer´s recommendation. The flow rate was set to 1.0 µL/min.

**Figure 1:**
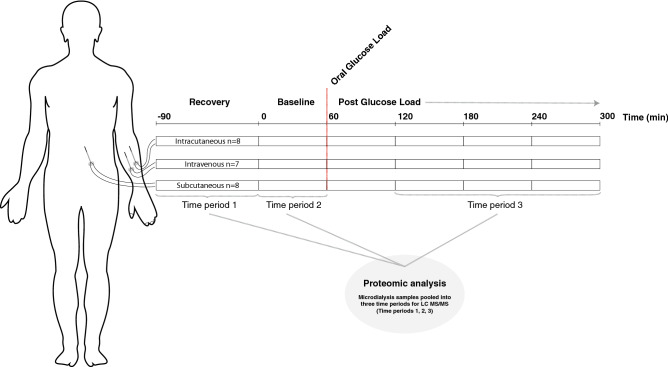
Experimental protocol and setup for in vivo microdialysis experiment in healthy subjects (N = 8). Microdialysate samples were pooled to reflect three time periods: (1) recovery period after catheter insertion, (2) baseline and (3) glucose provocation. The figure was designed in Adobe Illustrator by the authors.

A timeline and an overview of the protocol is shown in Fig. 1. After insertion of microdialysis catheter a resting period of 90 min called the recovery period was followed. A recovery period is commonly used in microdialysis studies to account for the local tissue trauma caused by the catheter insertion, and also to let the microdialysis system stabilize in the tissue. Microdialysis samples were collected with regular intervals of 15 min and ended at 300 min. The first 60 min after recovery period referred to baseline measurement. After the baseline period, subjects then performed an oral glucose tolerance test (OGTT) to increase the endogenous insulin production and samples collection was continued every 15 min. According to the WHO standard, the subjects ingested 75 g glucose (APL AB, Stockholm, Sweden) diluted in 2 dL water within 5 min. Lag time, the time it takes for the microdialysate to flow from the membrane to the microvial, was 5.1 min. To compensate for this, the oral glucose load was therefore ingested 5 min before the exchange of the last vial during baseline. Hence, no compensation for lag time was needed during data analysis.

Microdialysis sampling then followed for four hours after the oral glucose load. All microdialysis vials (M dialysis AB, Stockholm, Sweden) were weighed (CPA225D, Sartorius Weighing Technology GmbH, Goettingen, Germany) before and after sampling, to determine the volume of the recovered dialysate in each vial. Vials were changed every 15 min, (except for during the recovery period) and placed on ice to avoid protein degradation. To increase the sample volume and the total protein concentration, the microdialysate was pooled to reflect three different time periods: 1) recovery period after catheter insertion, 2) baseline, and 3) glucose provocation. For this proteomic study was 90 µl from time period 1, 10 µl from time period 2 and 30 µl from time period 3 available for proteomic analysis. Time period 3, “glucose provocation”, included dialysate from one to four hours after the oral glucose load was ingested. The dialysate from the first hour after the oral glucose load was excluded in order to create a distinct difference between the baseline period and the glucose provocation. The dialysate samples were then frozen at − 86 °C awaiting further analysis.

### Proteomics

#### Sample preparation

The dialysate samples contained a high amount of albumin since albumin was added to the perfusate as a colloid. High concentrations of albumin would interfere with the protein quantification and mask the identification of other proteins in LC–MS/MS. Each sample was therefore depleted of albumin using an albumin and IgG depletion column (Thermo Fisher Scientific, Pierce Biotechnology, Rockford, USA) according to the manufacturer´s recommendation. Briefly, each dialysate sample was added to the resin slurry in each column and then incubated in room temperature on a shaker for 30 min. The depleted microdialysate sample was collected by centrifuging the spin column for 2 min at 1000×g.

The samples were then desalted and concentrated using 3 kDa Amicon spin-filter (Merck Millipore, Darmstadt, Germany) according to the manufacturer’s recommendation. The concentrated samples were dried by speed vacuum concentrator (Savant, Farmingdale, NY, USA) and re-dissolved in 40 μL of urea buffer solution (8 M urea in 25 mM ammonium bicarbonate) to denature the proteins and incubated in room temperature for 2 h. The proteins were reduced with dithiothreitol (DTT) (25 mM) and incubated for 15 min, and then alkylated with iodoacetamide (75 mM) for an additional 15 min. Finally, the samples were diluted with 25 mM ammonium bicarbonate up to 250 μL and desalted. Protein concentration was then measured using 2D quant kit (GE Healthcare, Uppsala, Sweden) according to the manufacturer´s recommendation before trypsinization. A total amount of 10 µg protein was removed to a new tube and incubated with trypsin (0.2 µg/µl) in a ratio of 1:25 w/w trypsin/protein at 37 °C overnight. The tryptic peptides were dried in speed vacuum concentrator and stored at -20 °C until analysis. On the day of the analysis the samples were first dissolved in 100 µl formic acid (0.1%). Then, 10 µl was applied to liquid chromatography tandem mass spectrometry. During the sample preparation all the samples were stored on ice to prevent protease activation.

#### LC–MS/MS

The samples were analyzed using a liquid chromatography tandem mass spectrometry (EASY-nLC-MS/MS, Thermo Scientific, Waltham, MA, USA). Peptides were separated by reverse phase chromatography on a C18 pre column (20 mm × 100 μm) followed by a C18 column (100 mm × 75 μm) with particle size 5 μm (NanoSeparatoons, Nieuwkoop, Netherlands) at a flow rate of 300 nL/min. The gradient buffers contained 0.1% formic acid in water (buffer A) and 0.1% formic acid in acetonitrile (buffer B), and a linear gradient from 0 to 100% of buffer B was used for 90 min to separate the peptides. Automated online analyzes were performed with a LTQ Orbitrap Velos Pro hybrid mass spectrometer (Thermo Scientific, Waltham, MA, USA).

Raw files were searched using MaxQuant v. 1.5.8.3 (Max Planck Institute of Biochemistry, Martinsried, Germany) against Uniprot Human database (downloaded 2019). The following searching parameters were used: trypsin as digestion enzyme; maximum number of missed cleavages 2; fragment ion mass tolerance 0.50 Da; parent ion mass tolerance 6.0 ppm; fixed modification was carbamidomethylation of cysteine. Data were filtered at 1% false discovery rate. The label-free quantitative (LFQ) values were normalized in the software as described earlier^18^.

### Statistical analysis

Data in text is presented as means and SD, while data in Supplementary Figures (Fig. S7) are presented as means and SEM. Figures and statistics were performed using GraphPad Prism version 6.0 (GraphPad Software, San Diego, California, USA). The proteomic data set was analyzed using multivariate data analysis (MVDA) using SIMCA-P + (version 15.0; Sartorius Stedim Biotech, Umeå, Sweden) as previously described^19^ and according to Wheelock & Wheelock^20^. Briefly, Principal Component Analysis (PCA) was first applied for the check of multivariate outliers using score plots in combination with Hotelling’s T2 and distance to model in X-space, and then Orthogonal Partial Least Square discriminant analysis (OPLS-DA) was applied for the investigations of proteome differences between the compartments and the changes over time. In the OPLS-DA, variables (regressors) were considered important if the variable influence on projection (VIP) value was greater than one. VIP indicates the relevance of each X-variable pooled over all dimensions and Y-variables (the group of variables that best explain Y). R^2^ describes the goodness of fit—the fraction of sum of squares of all the variables explained by a principal component, and Q^2^ describes the goodness of prediction—the fraction of the total variation of the variables that can be predicted using principal component cross validation methods^21^. A difference between R^2^ and Q^2^ greater than 0.3 implies overfitting, meaning that the robustness of the model is poor. The more components the model consists of the higher the risk for “noise” contamination. More components would result in a higher R^2^ but only at the expense of a lower prediction score of the Q^2^. The validity of the model was estimated using cross validated analysis of variance (CV-ANOVA). A *p* value < 0.05 for CV-ANOVA indicate that the multivariate regressions were significant.

### Bioinformatics

The bioinformatic tool STRING (Search Tool for Retrieval of Interacting Genes/Proteins) was used to analyze the protein–protein association network^22^. Protein accession numbers (as described in UniProt) were entered in the search engine (multiple proteins) for (i) all detected significant proteins (VIP > 1) in each compartment (intracutaneous, subcutaneous and intravenous), (ii) the significant proteins (VIP > 1) identified as important for the separation between the different time periods in all compartments (Table 1), and (iii) the significant proteins (VIP > 1) identified as important for the separation between the compartments before and after glucose provocation (Table 2). Immunoglobulin kappa light chain and Immunoglobulin kappa constant can not be identified by the search engine and were therefore excluded in the STRING analysis. The following settings were applied: Organism Homo sapiens; the maximum number of interactions was query proteins only; interaction score was set to highest confidence (0.900); and an FDR (false discovery rate) ≤ 0.05 was used when classifying the Biological Process (GO) of each protein. For each obtained network, PPI enrichment *p* value was reported. In the network figures, each protein is represented by a colored node while protein–protein interaction and association are represented by a line. Higher combined confidence scores are represented by thicker lines.

**Table 1:** Significant proteins (VIP > 1) in the orthogonal partial least square discriminant analysis (OPLS-DA) model of all three compartments (intracutaneous, subcutaneous and intravenous) that contributed most to the separation between the different time periods.

Protein ID	Gene name	Protein name	Compartment	VIP
P02766	TTR	Transthyretin	Intracutaneous	1.37
P04217	A1BG	Alpha-1B-glycoprotein	Subcutaneous	1.37
P00450	CP	Ceruloplasmin	Subcutaneous	1.35
P02768	ALB	Albumin	Intracutaneous	1.35
P00738;P00739	HP	Haptoglobin/haptoglobin-related protein	Subcutaneous	1.32
P02652	APOA2	Apolipoprotein A-II	Intracutaneous	1.31
P02768	ALB	Albumin	Subcutaneous	1.30
P02763	ORM1	Alpha-1-acid glycoprotein 1	Subcutaneous	1.28
P01042	KNG1	Kininogen-1	Subcutaneous	1.28
P43652	AFM	Afamin	Intracutaneous	1.27
P02652	APOA2	Apolipoprotein A-II	Subcutaneous	1.24
P01011	AACT	Alpha-1-antichymotrypsin	Intravenous	1.24
P02763	ORM1	Alpha-1-acid glycoprotein 1	Intracutaneous	1.23
P02765	AHSG	Alpha-2-HS-glycoprotein	Intravenous	1.23
P02750	LRG1	Leucine-rich alpha-2-glycoprotein	Subcutaneous	1.23
P04217	A1BG	Alpha-1B-glycoprotein	Intracutaneous	1.22
P02766	TTR	Transthyretin	Subcutaneous	1.21
Q96PD5	PGLYRP2	N-acetylmuramoyl-L-alanine amidase	Subcutaneous	1.21
P01011	AACT	Alpha-1-antichymotrypsin	Subcutaneous	1.20
P68871	HBB	Hemoglobin subunit beta	Intracutaneous	1.20
P69905	HBA1	Hemoglobin subunit alpha	Intracutaneous	1.20
P02750	LRG1	Leucine-rich alpha-2-glycoprotein	Intracutaneous	1.20
P02768	ALB	Albumin	Intravenous	1.19
O75882	ATRN	Attractin	Intracutaneous	1.19
P01011	AACT	Alpha-1-antichymotrypsin	Intracutaneous	1.18
P00738;P00739	HP	Haptoglobin/haptoglobin-related protein	Intravenous	1.18
P02766	TTR	Transthyretin	Intravenous	1.15
P25311	AZGP1	Zinc-alpha-2-glycoprotein	Subcutaneous	1.15
P02765	AHSG	Alpha-2-HS-glycoprotein	Subcutaneous	1.15
P02753	RBP4	Retinol-binding protein 4	Intravenous	1.14
P00738;P00739	HP	Haptoglobin/haptoglobin-related protein	Intracutaneous	1.14
P19652	ORM2	Alpha-1-acid glycoprotein 2	Intracutaneous	1.14
P43251	BTD	Biotinidase	Intracutaneous	1.13
P68871	HBB	Hemoglobin subunit beta	Intravenous	1.13
P02765	AHSG	Alpha-2-HS-glycoprotein	Intracutaneous	1.12
P02760	AMBP	Protein AMBP	Intracutaneous	1.11
Q9NZP8	C1RL	Complement C1r subcomponent-like protein	Intracutaneous	1.11
P01019	AGT	Angiotensinogen	Intracutaneous	1.11
P25311	AZGP1	Zinc-alpha-2-glycoprotein	Intracutaneous	1.09
P02763	ORM1	Alpha-1-acid glycoprotein 1	Intravenous	1.09
P00450	CP	Ceruloplasmin	Intracutaneous	1.08
P01009	SERPINA1	Alpha-1-antitrypsin	Intravenous	1.08
P02749	APOH	Beta-2-glycoprotein 1	Intracutaneous	1.08
P01042	KNG1	Kininogen-1	Intracutaneous	1.07
P25311	AZGP1	Zinc-alpha-2-glycoprotein	Intravenous	1.06
P04217	A1BG	Alpha-1B-glycoprotein	Intravenous	1.06
P49454	CENPF	Centromere protein F	Subcutaneous	1.06
O75882	ATRN	Attractin	Intravenous	1.05
P01019	AGT	Angiotensinogen	Intravenous	1.05
Q96PD5	PGLYRP2	N-acetylmuramoyl-L-alanine amidase	Intracutaneous	1.05
Q9NZP8	C1RL	Complement C1r subcomponent-like protein	Subcutaneous	1.04
P02787	TF	Serotransferrin	Intracutaneous	1.04
P02787	TF	Serotransferrin	Subcutaneous	1.01
P02790	HPX	Hemopexin	Intravenous	1.00

Proteins were considered significant if VIP (variable influence of projection) value was > 1. Protein ID (accession number) and gene name are referred to according to the protein database Uniprot (http://uniprot.org).

**Table 2:** Significant proteins (VIP > 1) in the orthogonal partial least square discriminant analysis (OPLS-DA) model of all three compartments (intracutaneous, subcutaneous and intravenous) that contributed most to the separation in protein expression before (baseline) and after glucose provocation (Fig. 4).

Protein ID	Gene name	Protein name	Compartment	VIP	Alteration after glucose provocation vs baseline
P02652	APOA2	Apolipoprotein A-II	Intracutaneous	1.54	↑
Q9NZP8	C1RL	Complement C1r subcomponent-like protein	Intracutaneous	1.47	↑
Q96PD5	PGLYRP2	N-acetylmuramoyl-L-alanine amidase	Intracutaneous	1.47	↑
P43652	AFM	Afamin	Intracutaneous	1.46	↑
P04217	A1BG	Alpha-1B-glycoprotein	Intracutaneous	1.44	↑
P02750	LRG1	Leucine-rich alpha-2-glycoprotein	Intracutaneous	1.41	↑
P02765	AHSG	Alpha-2-HS-glycoprotein	Intracutaneous	1.39	↑
P02768	ALB	Albumin	Subcutaneous	1.38	↑
Q96PD5	PGLYRP2	N-acetylmuramoyl-L-alanine amidase	Subcutaneous	1.37	↑
P02768	ALB	Albumin	Intracutaneous	1.36	↑
P00738;P00739	HP	Haptoglobin/haptoglobin-related protein	Intravenous	1.35	↑
P01019	AGT	Angiotensinogen	Intracutaneous	1.34	↑
P68871	HBB	Hemoglobin subunit beta	Intravenous	1.34	↑
P04217	A1BG	Alpha-1B-glycoprotein	Intravenous	1.34	↑
P04217	A1BG	Alpha-1B-glycoprotein	Subcutaneous	1.33	↑
P02766	TTR	Transthyretin	Intracutaneous	1.33	↓
P43251	BTD	Biotinidase	Intracutaneous	1.32	↑
P02652	APOA2	Apolipoprotein A-II	Subcutaneous	1.30	↑
P02765	AHSG	Alpha-2-HS-glycoprotein	Intravenous	1.30	↑
P01019	AGT	Angiotensinogen	Subcutaneous	1.29	↑
P01019	AGT	Angiotensinogen	Intravenous	1.29	↑
P43652	AFM	Afamin	Intravenous	1.27	↑
P00738;P00739	HP	Haptoglobin/haptoglobin-related protein	Intracutaneous	1.27	↑
P02763	ORM1	Alpha-1-acid glycoprotein 1	Intracutaneous	1.27	↓
P02750	LRG1	Leucine-rich alpha-2-glycoprotein	Subcutaneous	1.26	↑
P00450	CP	Ceruloplasmin	Subcutaneous	1.25	↑
P01011	AACT	Alpha-1-antichymotrypsin	Subcutaneous	1.23	↓
P02765	AHSG	Alpha-2-HS-glycoprotein	Subcutaneous	1.22	↑
P02750	LRG1	Leucine-rich alpha-2-glycoprotein	Intravenous	1.22	↑
P02760	AMBP	Protein AMBP	Intracutaneous	1.22	↑
P02749	APOH	Beta-2-glycoprotein 1	Intracutaneous	1.21	↑
P01011	AACT	Alpha-1-antichymotrypsin	Intracutaneous	1.21	↓
P68871	HBB	Hemoglobin subunit beta	Intracutaneous	1.20	↑
P00738;P00739	HP	Haptoglobin/haptoglobin-related protein	Subcutaneous	1.19	↑
P01008	SERPINC1	Antithrombin-III	Intravenous	1.19	↓
P02763	ORM1	Alpha-1-acid glycoprotein 1	Subcutaneous	1.17	↓
P01042	KNG1	Kininogen-1	Subcutaneous	1.15	↑
P01042	KNG1	Kininogen-1	Intracutaneous	1.15	↑
P19652	ORM2	Alpha-1-acid glycoprotein 2	Intracutaneous	1.15	↓
P00450	CP	Ceruloplasmin	Intracutaneous	1.14	↑
P69905	HBA1	Hemoglobin subunit alpha	Intravenous	1.14	↑
P02753	RBP4	Retinol-binding protein 4	Intracutaneous	1.13	↑
P02768	ALB	Albumin	Intravenous	1.13	↑
P05155	SERPING1	Plasma protease C1 inhibitor	Intravenous	1.08	↑
Q9NZP8	C1RL	Complement C1r subcomponent-like protein	Subcutaneous	1.06	↑
P01011	AACT	Alpha-1-antichymotrypsin	Intravenous	1.05	↓
Q96PD5	PGLYRP2	N-acetylmuramoyl-L-alanine amidase	Intravenous	1.04	↑
P68871	HBB	Hemoglobin subunit beta	Subcutaneous	1.04	↑
O75882	ATRN	Attractin	Intravenous	1.03	↑
P25311	AZGP1	Zinc-alpha-2-glycoprotein	Intracutaneous	1.02	↓
P01042	KNG1	Kininogen-1	Intravenous	1.02	↑
P02753	RBP4	Retinol-binding protein 4	Intravenous	1.02	↓
P43251	BTD	Biotinidase	Subcutaneous	1.01	↑

↑ = Upregulated; ↓ = Downregulated after glucose provocation compared to before (baseline). Proteins were considered significant if VIP (variable influence of projection) value was > 1. Protein ID (accession number) and gene name are referred to according to the protein database Uniprot (http://uniprot.org).

## Results

### Overview

Due to technical issues during catheter insertion, one of the intravenous microdialysis catheters was never inserted. The other 23 catheters were successfully inserted, and adequate volume recovery was obtained in all microvials (intracutaneous: 95 ± 5%; subcutaneous 96 ± 4%; intravenous 99 ± 5%). This resulted in a total of 69 dialysate samples, but one sample was unfortunately spoiled during albumin depletion. In the remaining 68 samples, mean protein concentration in the intracutaneous samples was 4.6 ± 4.0 µg/µL, 4.4 ± 4.5 µg/µL in the subcutaneous samples and 1.4 ± 1.7 µg/µL in the intravenous samples. Due technical issues during LC–MS/MS, two samples from the subcutaneous compartment and three from the intravenous compartment were excluded. Detailed numeric data can be found in the supplementary files.

### Proteomic analysis

**Figure 2:**
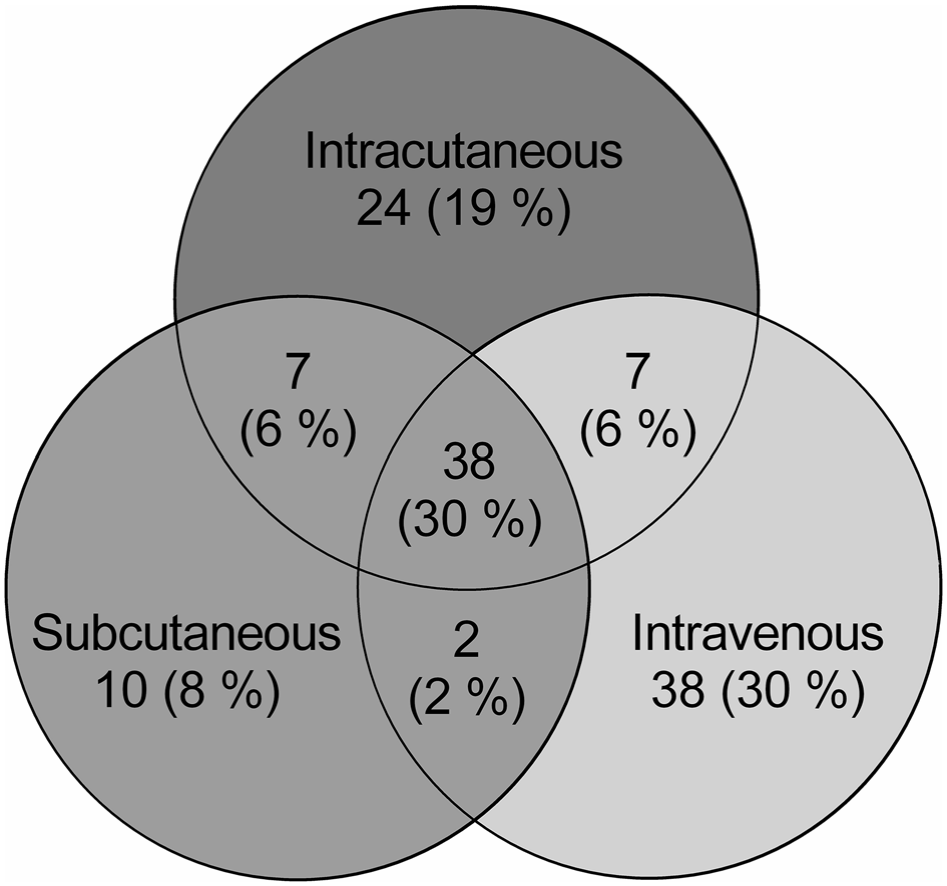
Three-way Venn diagram visualizing the distribution of proteins between the different tissue compartments; intracutaneous, subcutaneous and intravenous.

A total of 126 proteins were detected in the microdialysate samples using LC–MS/MS, where 31% of the proteins could be found in all three tissue compartments. The distribution of the detected proteins in each compartment is depicted in the Venn diagram (Fig. 2). Seven proteins (apolipoprotein D, carbonic anhydrase 1, hemoglobin subunit delta, hornerin, keratin type II cytoskeletal 1, keratin type II cytoskeletal 2 epidermal, rho guanine nucleotide exchange factor 9) were found only in the intracutaneous and subcutaneous compartments. In the intracutaneous and intravenous compartments another seven proteins overlapped (actin, clusterin, complement C3, fibrinogen beta chain, homeobox protein Hox-B3, thymosin beta-4, vasorin), while only two proteins were found to be unique for the intravenous and subcutaneous compartments (alpha-2-macroglobulin, inter-alpha-trypsin inhibitor heavy chain H4). A detailed list with names of all detected proteins in each tissue compartment is presented in Supplementary Table S1.

#### Protein changes over time in each compartment

Multivariate statistical analysis was performed to investigate the differences in protein expression between the different time periods; (1) recovery period after catheter insertion, (2) baseline, and (3) glucose provocation, in all three respective compartments; intracutaneous, subcutaneous and intravenous.

##### Intracutaneous

The unsupervised PCA model did not identify any strong outliers. The OPLS-DA model consisted of one predictive and one orthogonal component (R^2^ = 0.49, Q^2^ = 0.42 and CV-ANOVA *p* value: 0.9E^−3^). A clear separation between the different time periods based on the proteome profile were found (see Supplementary Fig. S1), where 28 proteins with VIP > 1 contributed most to the separation.

##### Subcutaneous

No strong outliers were identified in the PCA model. The OPLS-DA model showed a good fit (R^2^ = 0.65), predictivity (Q^2^ = 0.55) and a highly significant CV-ANOVA (*p* value = 1.1E^−5^). The subcutaneous OPLS-DA model, consisting of one predictive and one orthogonal component, also showed a clear separation between the different time periods based on the proteome profile (see Supplementary Fig. S2).

#### Intravenous

No strong outliers were identified in the intravenous PCA model. The OPLS-DA model, consisting of one predictive and one orthogonal component, showed a clear separation between the different time periods based on the proteome profile (see Supplementary Fig. S3). The intravenous OPLS-DA model showed a good fit (R^2^ = 0.42) and predictivity (Q^2^ = 0.35), however not statistically significant (CV-ANOVA *p* value = 0.10).

#### Protein changes between all compartments (in all time periods)

**Figure 3:**
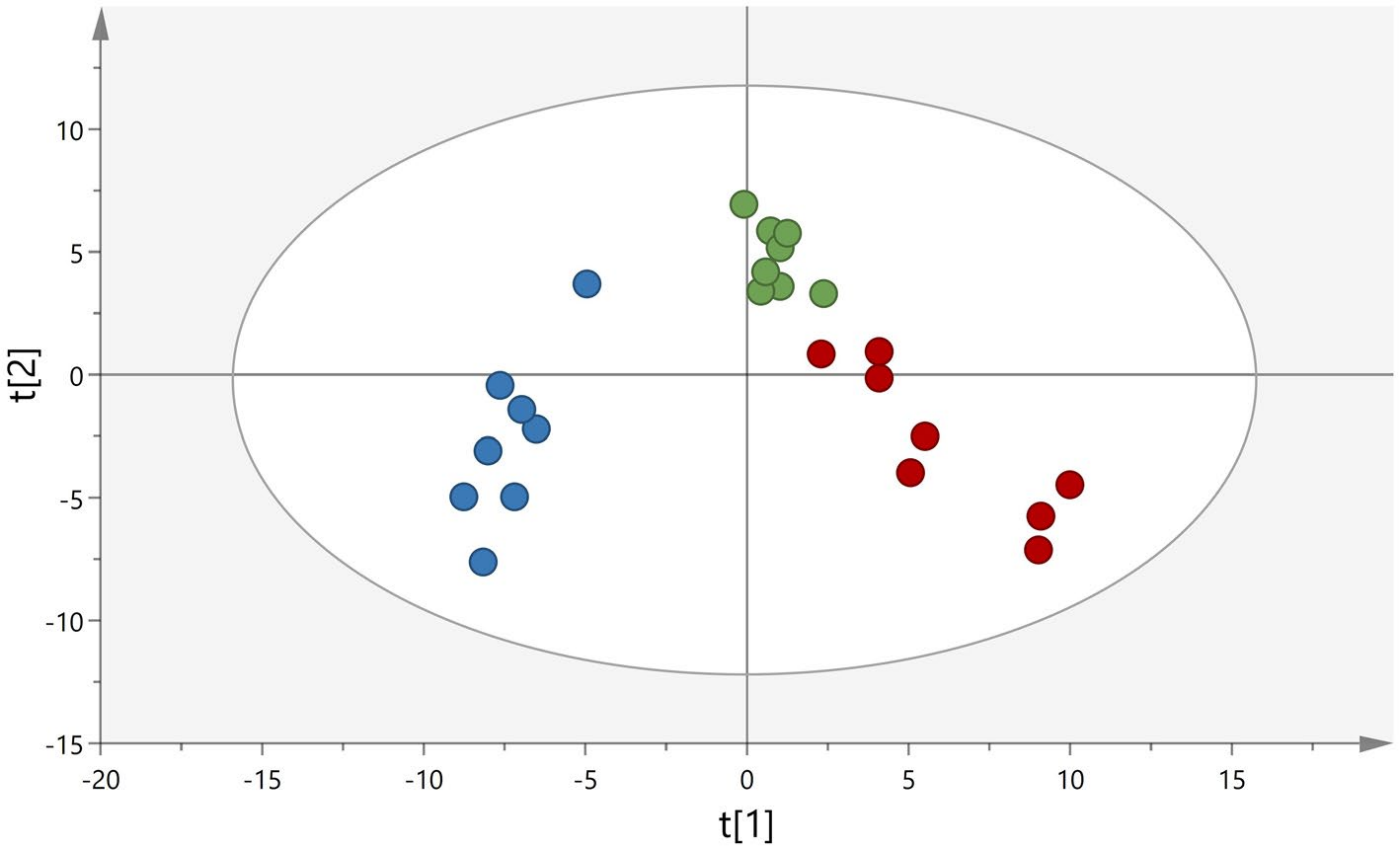
Principal component analysis (PCA) of the proteome over time. The score plot shows the dynamic changes of protein expression in all compartments; intracutaneous (green), subcutaneous (blue) and intravenous (red). The x-axis represents intergroup differences while the y-axis represents intragroup differences.

To investigate the dynamic changes of the protein patterns (in time period 1–3) in all compartments (intracutaneous, subcutaneous and intravenous) multivariate statistical analysis was performed. No strong outliers were identified in the unsupervised PCA models and, as shown in Fig. 3, a distinct separation between the different time periods was already detected in this unsupervised model.

The OPLS-DA model consisted of two predictive components and showed a clear separation between the time periods based on the proteome profile (R^2^ = 0.83, Q^2^ = 0.79, *p*-value = 1.1E^−10^). Table 1 presents the 54 proteins from all three compartments with VIP > 1 that contributed most to the separation between the different time periods, where 24 originated from the intracutaneous compartment, 16 from the subcutaneous compartment and 14 from the intravenous compartment. The highest VIP values, i.e. the proteins contributing most to the separation between the different time periods, were found in the intracutaneous and the subcutaneous compartments. A detailed list with LFQ intensity for each protein in Table 1 is presented in Supplementary Table S2.

#### Protein changes between all compartments before and after glucose provocation (time period 2 and 3)

**Figure 4:**
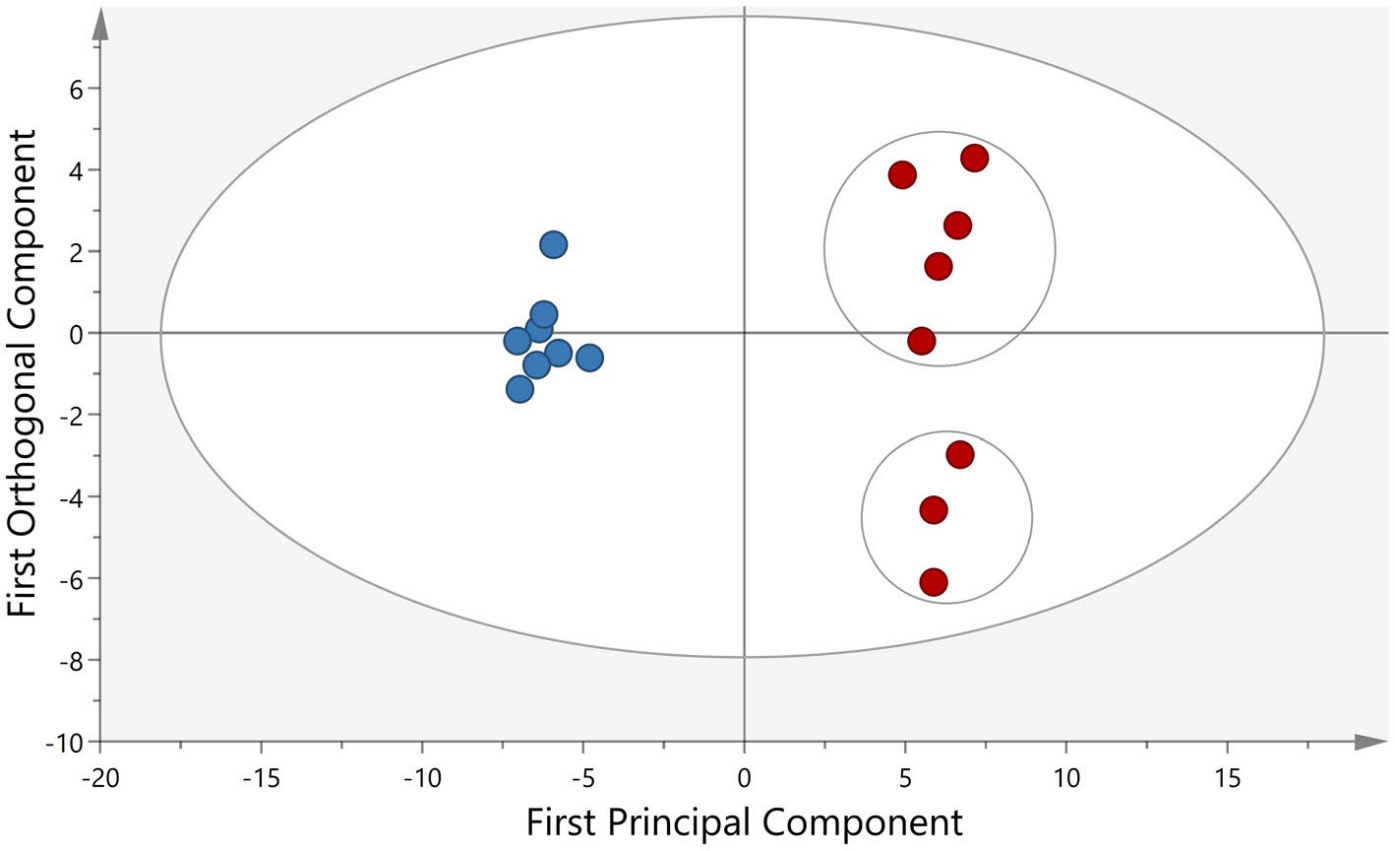
Orthogonal partial least square discriminant analysis (OPLS-DA) of the proteome before and after glucose provocation. The score plot shows the separation in protein expression between baseline (blue) and after glucose provocation (red). R^2^ = 0.99, Q^2^ = 0.91, CV-ANOVA *p* value = 1.6E^−10^. The x-axis represents intergroup differences while the Y-axis represents intragroup differences.

To investigate protein biomarkers that might affect the microcirculation following a glucose provocation, multivariate statistical analysis was performed in all compartments (intracutaneous, subcutaneous and intravenous), but only during time period 2 and 3 (i.e. before and after glucose provocation. The OPLS-DA model (Fig. 4) consisted of one predictive and one orthogonal component, and showed a clear separation of the proteome profile before and after glucose provocation (R^2^ = 0.99, Q^2^ = 0.91, *p*-value = 1.6E^−5^), where the 53 proteins contributing most to the separation (VIP > 1) is presented in Table 2. Two subgroups, marked with circles (Fig. 4), were identified in the proteome after glucose provocation. A detailed list with LFQ intensity for each protein in Table 2 is presented in Supplementary Table S3.

### Bioinformatics

#### Protein changes over time in each compartment

The significant proteins (VIP > 1) from the OPLS-DA model from the respective compartments (intracutaneous, subcutaneous and intravenous; i.e. from Supplementary Figs. S1, S2, S3) were analyzed to investigate the protein–protein association network using STRING analysis. These STRING analyzes is presented in Supplementary Figs. S4, S5, S6. The proteins interesting for the aim of this study were involved in the biological process of vascular actions (vasodilation, regulation of blood vessel size, positive regulation of nitric oxide biosynthetic processes and regulation of cellular response to insulin stimulus), where interactions were found between four proteins intracutaneously and subcutaneously (angiotensinogen, kininogen-1, alpha-2-HS-glycoprotein and hemoglobin subunit beta) and five intravenously (angiotensinogen, alpha-2-HS-glycoprotein and hemoglobin subunit beta, fibrinogen alpha chain, fibrinogen gamma chain). PPI enrichment for all networks in STRING were < 1.0E^−16^.

#### Protein changes between all compartments (in all time periods)

**Figure 5:**
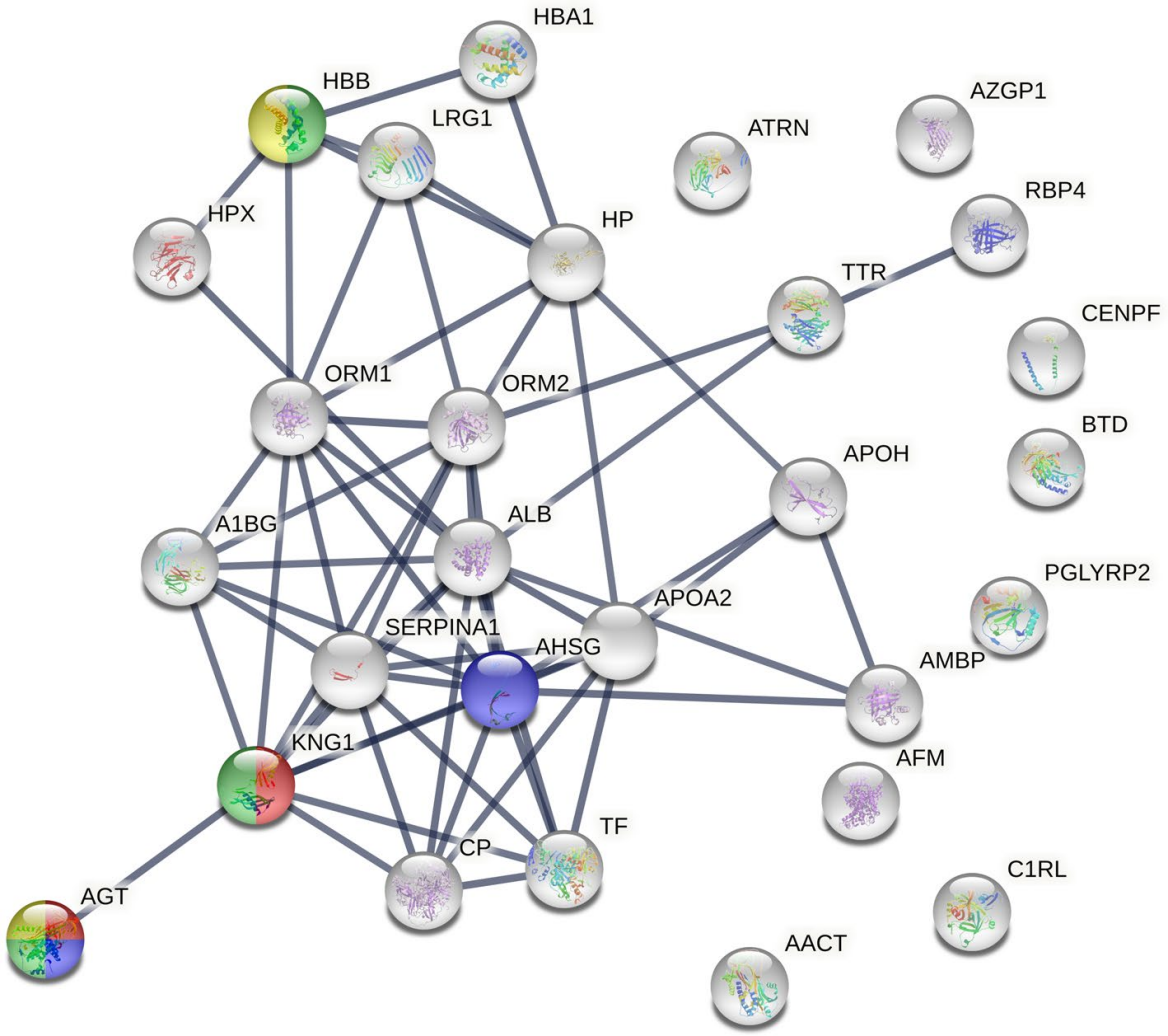
Pathway analysis of proteins associated with vascular actions of the proteins most important for the separation between the time periods in all compartments (intracutaneous, subcutaneous and intravenous) using the STRING database. Vascular pathways highlighted: red = vasodilation, blue = regulation of cellular response to insulin stimulus, green = regulation of blood vessel diameter, yellow = positive regulation of nitric oxide biosynthetic process. PPI enrichment was < 1.0E^−16^. Proteins involved in vascular pathways: AGT = Angiotensinogen, KNG = Kininogen-1, AHSG = Alpha-2-HS-glycoprotein, HBB = Hemoglobin subunit beta.

The proteins contributing most to the separation between the compartments during the whole experiment (Table 1) were also analyzed using STRING. Figure 5 shows the interactions between proteins with vascular effects, where four proteins (angiotensinogen, kininogen-1, alpha-2-HS-glycoprotein and hemoglobin subunit beta) were found. LFQ intensity for these proteins are shown in Supplementary Fig. S7. PPI enrichment was < 1.0E^−16^.

#### Protein changes between all compartments before and after glucose provocation (time period 2 and 3)

**Figure 6:**
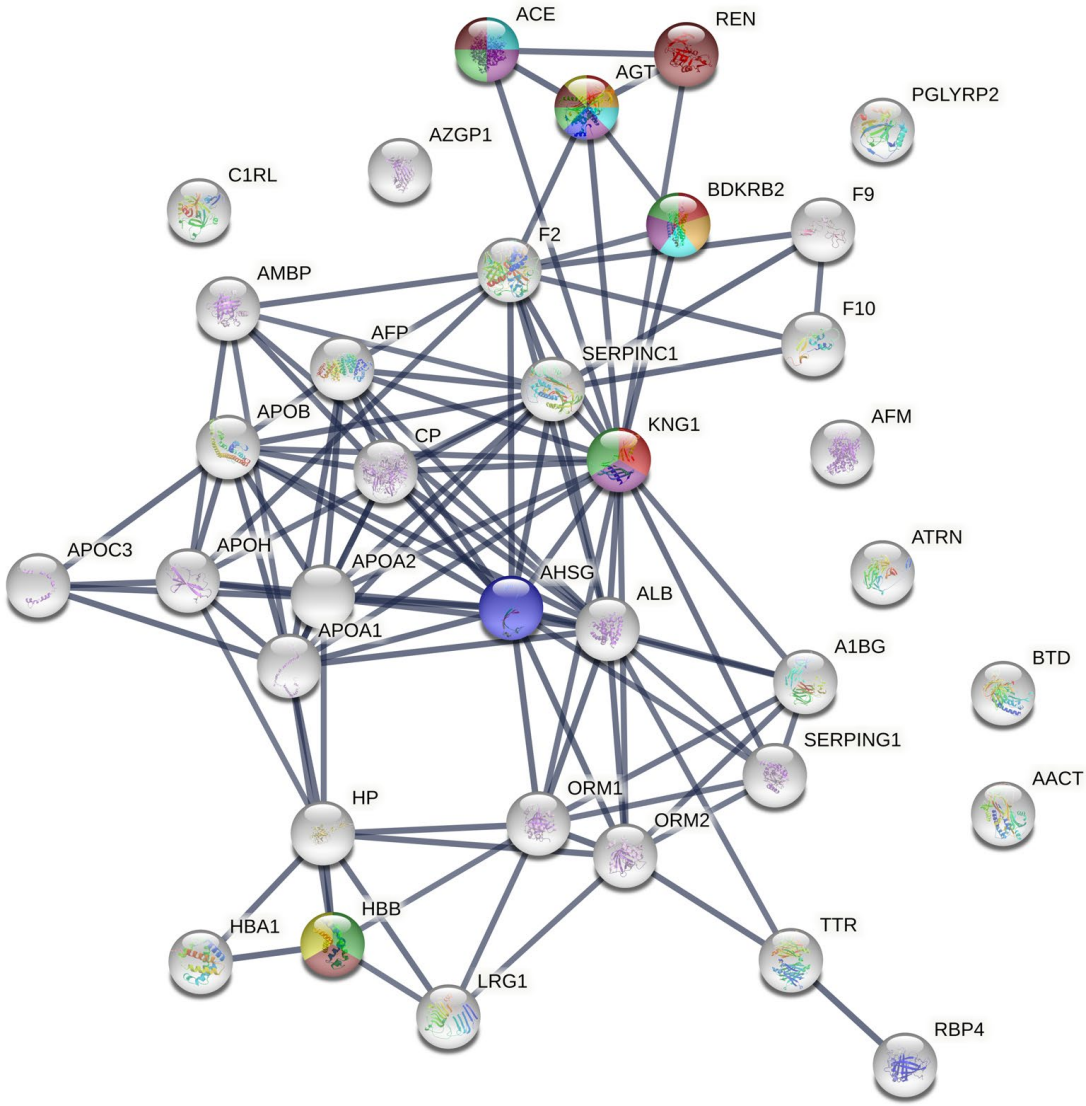
Extended pathway analysis of proteins associated with vascular actions of the proteins most important for the separation before (baseline) and after glucose provocation using the STRING database. Vascular pathways highlighted: red = vasodilation, blue = regulation of cellular response to insulin stimulus, green = regulation of blood vessel diameter, yellow = positive regulation of nitric oxide biosynthetic process, turquoise = regulation of vasoconstriction, orange = vasoconstriction, purple = regulation of tube diameter, brown = regulation of blood pressure. PPI enrichment was < 1.0E^−16^. Proteins involved in vascular pathways: AGT = Angiotensinogen, KNG = Kininogen-1, AHSG = Alpha-2-HS-glycoprotein, HBB = Hemoglobin subunit beta, ACE = Angiotensin converting enzyme, REN = Renin, BDKRB2 = B2 bradykinin receptor.

Supplementary Fig. S8 presents the protein–protein interactions between the proteins in Table 2, i.e. the proteins most important for the separation before and after glucose provocation. When adding associated proteins to see larger connections of proteins involved in larger biological systems, further interactions was found, comprising renin, angiotensin converting enzyme and B2 bradykinin receptor (Fig. 6).

## Discussion

The hormone insulin is secreted in the healthy body as a reaction to glucose ingestion. Its metabolic effects in peripheral tissue are well investigated, however it is less known what pathways orchestrate its effects on local tissue microcirculation. This study therefore used an innovative approach of combining microdialysis and tandem mass spectrometry to investigate changes in the human proteome in the skin and surrounding tissues after an oral glucose provocation. Microdialysate samples from the intracutaneous, subcutaneous and intravenous compartments revealed a total of 126 proteins, whereof 34% were found in both compartments. There was a relatively distinct variation in protein expression over time (Table 1), and after glucose provocation (Table 2), which was most pronounced in the intracutaneous and subcutaneous compartments, compared to the intravenous compartment. Most relevant from the perspective of local microcirculatory physiological functions was the strong association of the four proteins; Angiotensinogen (AGT), kininogen-1 (KNG1), alpha-2-HS-glycoprotein (AHSG) and hemoglobin subunit beta (HBB). They were all upregulated after glucose provocation compared to baseline (Supplementary Fig. S7). Previous research on how the expression of these proteins is affected by a glucose provocation is limited, although the connection between especially angiotensinogen and kininogen-1 is well known in relation to microcirculatory processes and effects on blood flow related to activation of the nitric oxide pathway. An improved understanding of what biological processes occur in skin and subcutaneous tissue, secondary to increased systemic insulin levels, is likely to be of importance for targeted treatments where glucose delivery to the target tissue is hampered as a result of serious illness and deranged glucose-insulin functions^23^.

Kininogen-1 is a precursor for kinins and plays an important role in the kallikrein-kinin system in the blood coagulation cascade^24,25^. Kininogen-1 is proteolytically cleaved into the active peptide bradykinin, a potent but short-lived vasoactive mediator. Bradykinin is proinflammatory (inducing increased vascular permeability and stimulates nociceptors), exert vasodilatory effects and is involved in glucose homeostasis (decreases blood glucose concentration)^24–27^. Bradykinin then acts on endothelial cell bradykinin B2 receptors to form nitric oxide^28^.

Angiotensinogen is the only known precursor to the renin-angiotensin system (RAS), which is an essential regulatory system for arterial blood pressure, vasoconstriction, body fluid and electrolyte homeostasis and proinflammatory processes^29–31^. Angiotensinogen itself mainly inhabits vasoconstrictive properties^29,31^ and is known to affect the development of vascular diseases such as hypertension and atherosclerosis^32,33^. Angiotensin II is formed after enzymatic cleavage of angiotensinogen, via angiotensin I. When acting through angiotensin 1 receptors (AT1-R), angiotensin II is a strong vasoconstrictor involved in development of hypertension, atherosclerosis, diabetes, and cardiac and kidney failure^34^. In contrast does angiotensin II also produce a vasodilator effect through the angiotensin 2 receptors (AT2-R) via the bradykinin dependent activation of endothelial nitric oxide (NO) synthase, which is believed to be a compensatory mechanism in order to protect the vessels from mechanical overload (too much vasoconstriction)^28,34^.

This interaction between the RAS system and the kallikrein-kinin system is visually shown in Fig. 6. Both these systems induce nitric oxide dependent vasodilation. Insulin is also known to induce vasodilation and increase glucose metabolism the nitric oxide pathway^8–10^. Angiotensinogen, amongst hundreds of other adipokines, are known to modulate insulin sensitivity and influence glucose/fat metabolism and obesity^35–37^. Schorr et al. showed that the insulin response to an oral glucose load was significantly related to increased plasma levels of angiotensinogen, which positively correlated with increased blood pressure^33^. Insulin treatment induces the expression of angiotensinogen in adipose tissue^37^. Elevated expressions of kininogen-1 respectively prekallikrein (cleaves kininogen to vasoactive peptide bradykinin) have also previously been described in type 1 diabetic patients and rats^38–40^, which in rats further could be reversed with insulin treatment^40^.

In 2004, Schremmer-Danninger et al. showed that kinin-B1 receptors (receptors for bradykinin) were upregulated in skin biopsies obtained following surgery, suggesting an important role of kinin-B1 receptors during the first phase of inflammation following injury^41^. Kininogen-1 was also upregulated in epidermis in skin biopsies following surgery. One could speculate that the microdialysis catheter insertion would trigger an upregulation of kininogen-1 in this current study. As shown in the LFQ-intensity of kininogen-1 in Supplementary Fig. S7B this was however not the case, whereas kininogen-1 was upregulated after glucose provocation.

In this study, angiotensinogen, but no other part of the RAS system, was found in all three tissue compartments. It has however previously been shown that the complete renin– angiotensin system (receptors for angiotensin I and angiotensin II, renin, angiotensinogen and angiotensin-converting enzyme) is present in human skin^42^. Presumably, the whole RAS system plays a role in the normal cutaneous homeostasis, as well as wound healing. Likewise has the whole kallikrein-kinin system previously been found intracutaneously^41^.

In addition to angiotensinogen and kininogen-1, hemoglobin subunit beta and alpha-2-HS-glycoprotein were also detected and are both known to have vascular effects. Hemoglobin subunit alpha and beta in the intracutaneous and subcutaneous compartments were upregulated only during time period 1, i.e. recovery after the catheter insertion. We interpret this as a minor local bleed, due to the catheter insertion, that however was not detected visually in the microdialysate. This was however not seen in the intravenous compartment, which could be explained by the higher laminar blood flow in the blood vessel. Alpha-2-HS-glycoprotein, also known as fetuin-A, has no direct effect on the microcirculation or the blood flow, but has indirect effects via regulation of insulin^43^. It is highly associated with insulin resistance, obesity type 2 diabetes and non-alcoholic fatty liver disease^44–47^. Alpha-2-HS-glycoprotein is an inhibitor of insulin receptor tyrosine kinase in the liver and skeletal muscle, thereby inhibiting the autophosphorylation of tyrosine kinase in insulin receptor and insulin receptor substrate 1 (IRS-1) resulting in insulin resistance in humans^48^.

Figure 4 shows a clear separation of proteome profile before and after glucose provocation. It is evident that the proteome is more homogenous at baseline compared to after glucose provocation, indicating that there is a biological variation in how the subjects respond to the provocation. Two subgroups were identified after glucose provocation, marked with circles. Same tendency to subgroup (including the same subjects who deviated in Fig. 4) was also seen in Fig. 3 and Supplementary Fig. S3. No apparent explanation can however be found in the demographic data of these subjects. Thus, this indicates a biological difference in the response, which should be further investigating before performing studies on insulin resistant patients.

Endothelial dysfunction affects the microcirculation and it would therefore be of great interest to study the differences in protein expression after glucose provocation from this study of healthy subjects to a group of insulin resistant patients in the future. This comparison could hopefully shed light on how the skin tissue response differs between healthy subjects and patients with insulin-dependent diabetes mellitus.

All analyzes in this study are performed with a microvascular perspective. A proteomic study like this generates an enormous amount of data. In a future study it would be interesting to reuse the material from this study to investigate other proteins involved in for example metabolic processes.

### Limitations

A great advantage with the combination of microdialysis and mass spectrometry is the possibility to study different target tissues dynamically, which cannot be achieved with sequential biopsies or other discontinuous sampling methods. There are however some limitations. The low concentration of proteins in the microdialysate samples makes identification of less abundant components difficult. It is also possible that some proteins were removed during albumin depletion. Since albumin needs to be added as a colloid to the microdialysate perfusate this step was however crucial, otherwise the albumin would have blocked the identification of other less abundant proteins. Another limitation of this pilot study is that we have not used additional methods to verify the findings. Therefore, further studies with a larger sample size are warranted to verify the results.

Furthermore, the proteins adsorbed to the microdialysis membrane have not been taken into consideration in the present study. It is highly likely that the membranes could provide further information of the microcirculatory biomarkers from these compartments following a glucose provocation.

## Conclusion

In summary, glucose provocation is known to cause insulin induced vasodilation through the nitric oxide pathway, and this study indicates that this is facilitated through the interactions of the RAS (angiotensinogen) and kallikrein-kinin (kininogen-1) systems.

## Supplementary Information

Supplementary Information 1.

Supplementary Information 2.

Supplementary Information 3.

Supplementary Information 4.

Supplementary Information 5.

Supplementary Information 6.

Supplementary Information 7.

Supplementary Information 8.

Supplementary Legends.

Supplementary Information 10.

Supplementary Information 11.

Supplementary Information 12.

Supplementary Information 13.

## Abbreviations

ELISA: Enzyme-linked immunosorbent assay
LFQ: Label-free quantification
LC–MS/MS: Liquid chromatography tandem mass spectrometry
OGTT: Oral glucose tolerance test
PCA: Principal component analysis
OPLS-DA: Orthogonal partial least squares discriminant analysis
SD: Standard deviation
SEM: Standard error of the mean
STRING: Search tool for retrieval of interacting genes/proteins
VIP: Variable importance in projection
