# Supplementary figures and images for: Investigation of proteins important for microcirculation using in vivo microdialysis after glucose provocation: a proteomic study
Source: Sci Rep. 2021 Sep 27;11:19093. doi: 10.1038/s41598-021-98672-8 (PMC8476624; doi:10.1038/s41598-021-98672-8)

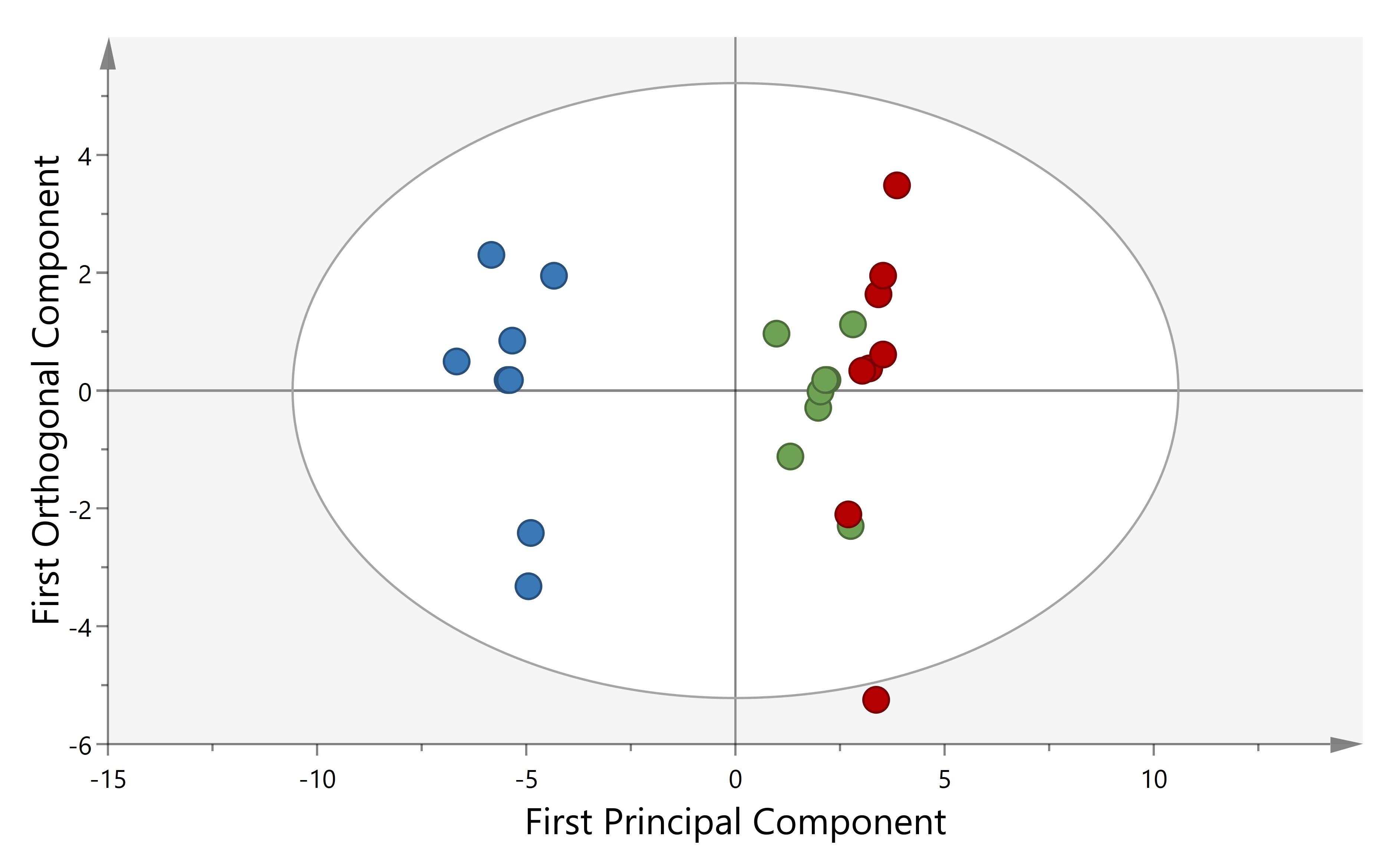

Supplement: Supplementary file 1 — Supplementary Information 1. [file 41598_2021_98672_MOESM1_ESM.jpg]

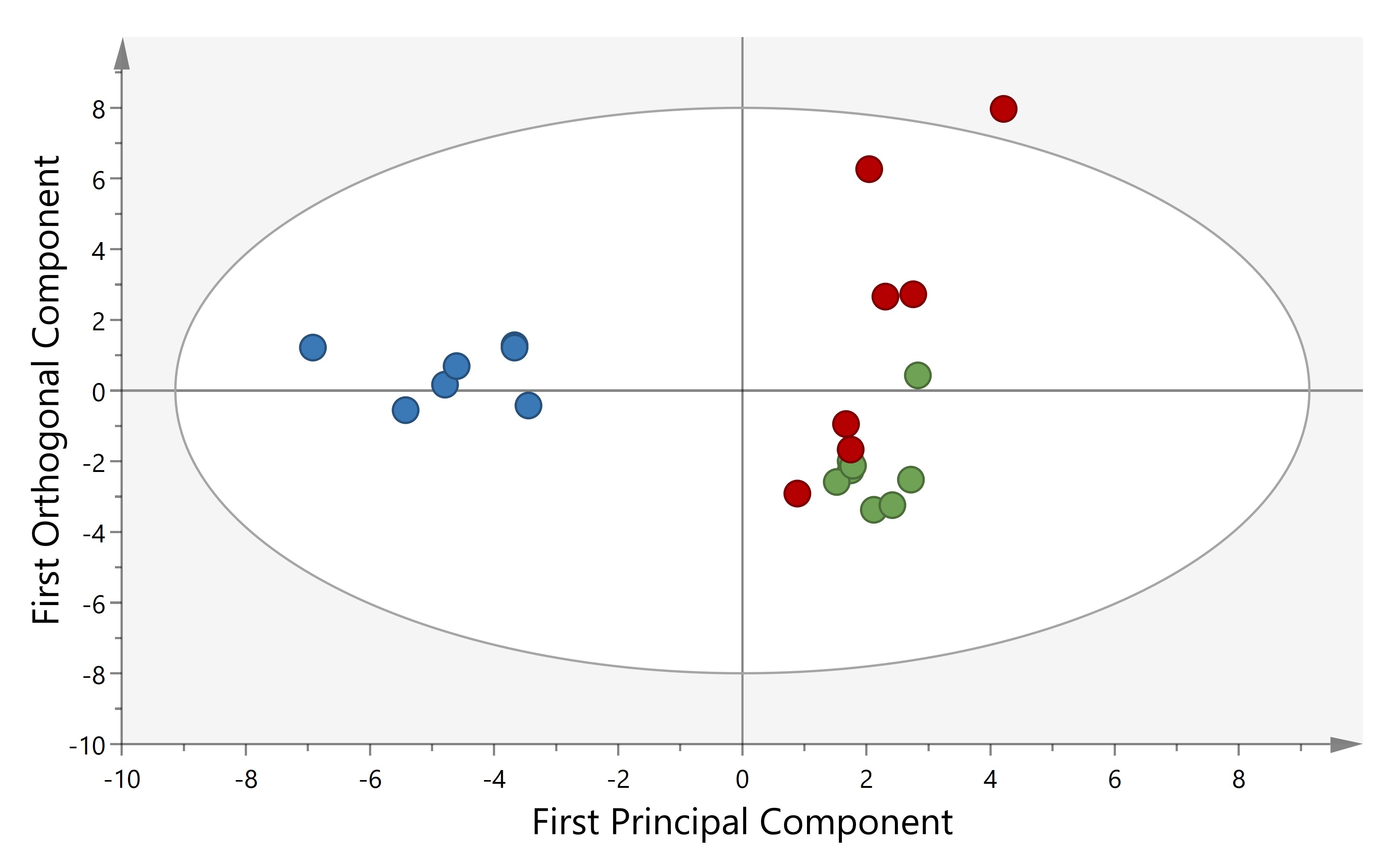

Supplement: Supplementary file 2 — Supplementary Information 2. [file 41598_2021_98672_MOESM2_ESM.jpg]

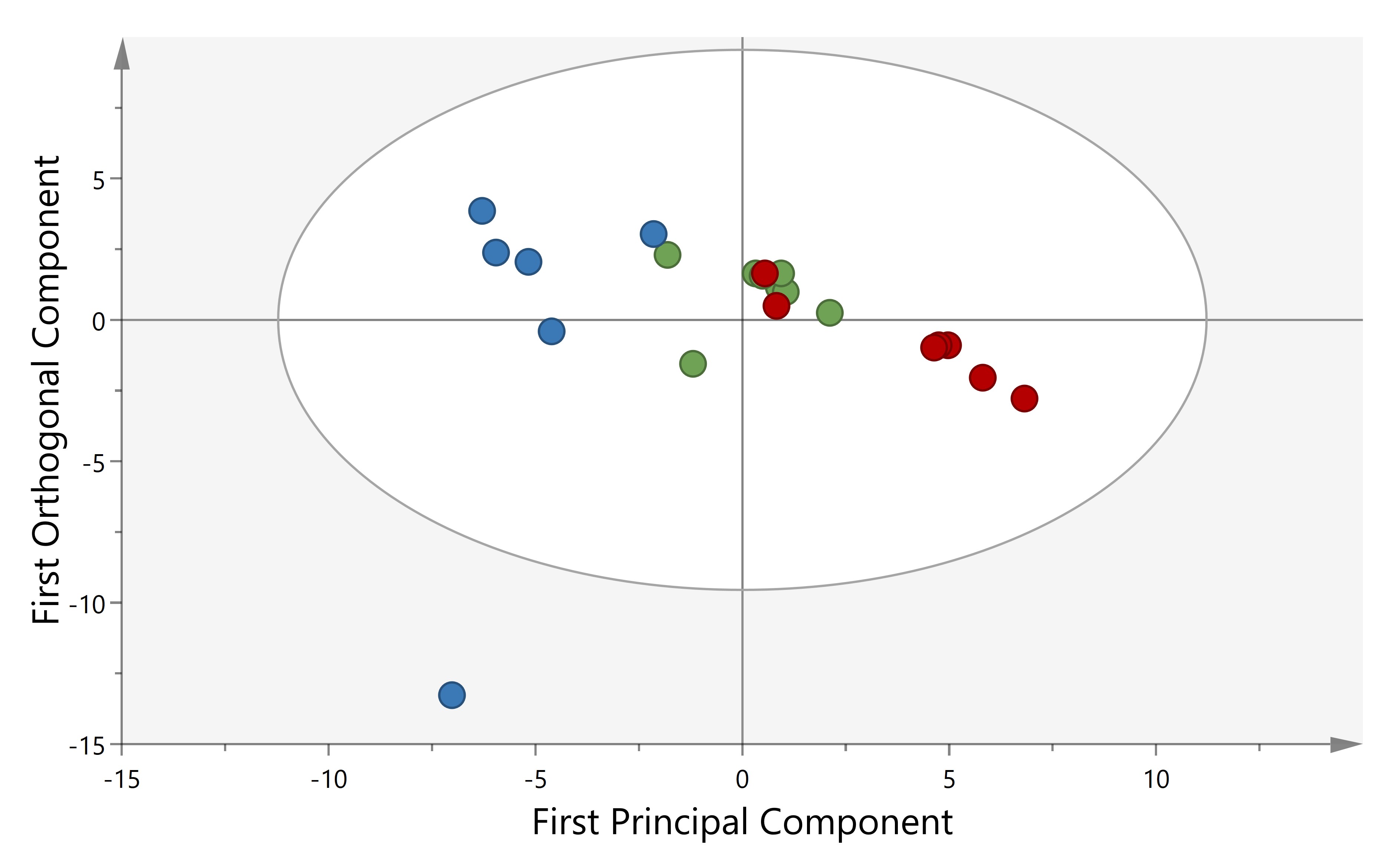

Supplement: Supplementary file 3 — Supplementary Information 3. [file 41598_2021_98672_MOESM3_ESM.jpg]

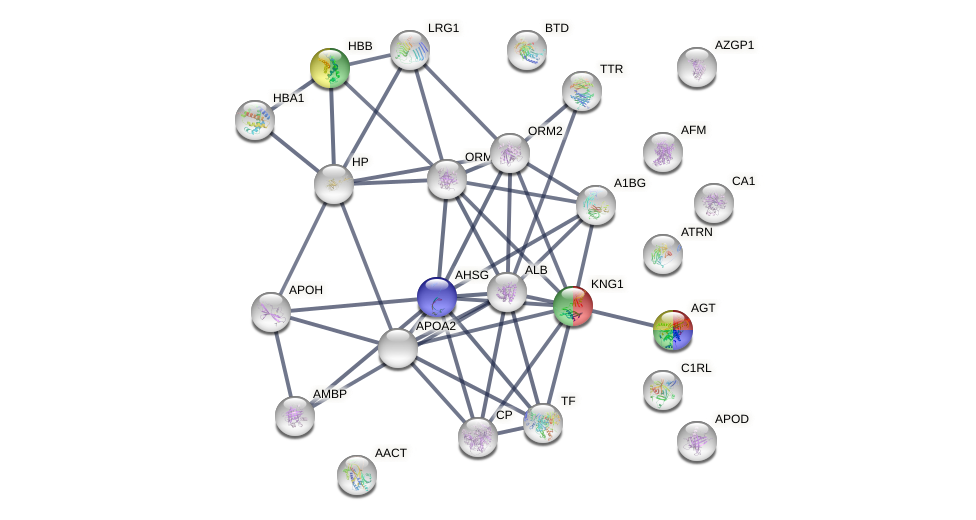

Supplement: Supplementary file 4 — Supplementary Information 4. [file 41598_2021_98672_MOESM4_ESM.png]

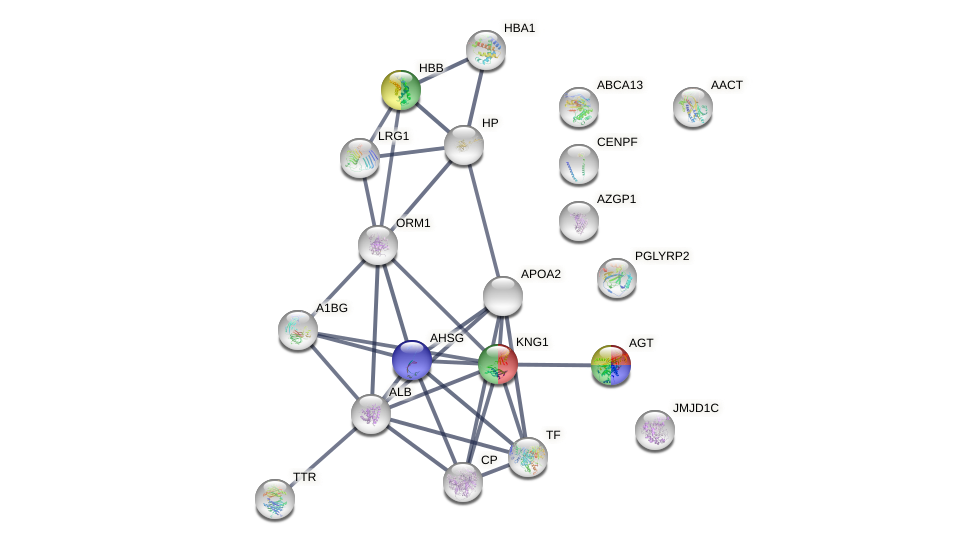

Supplement: Supplementary file 5 — Supplementary Information 5. [file 41598_2021_98672_MOESM5_ESM.png]

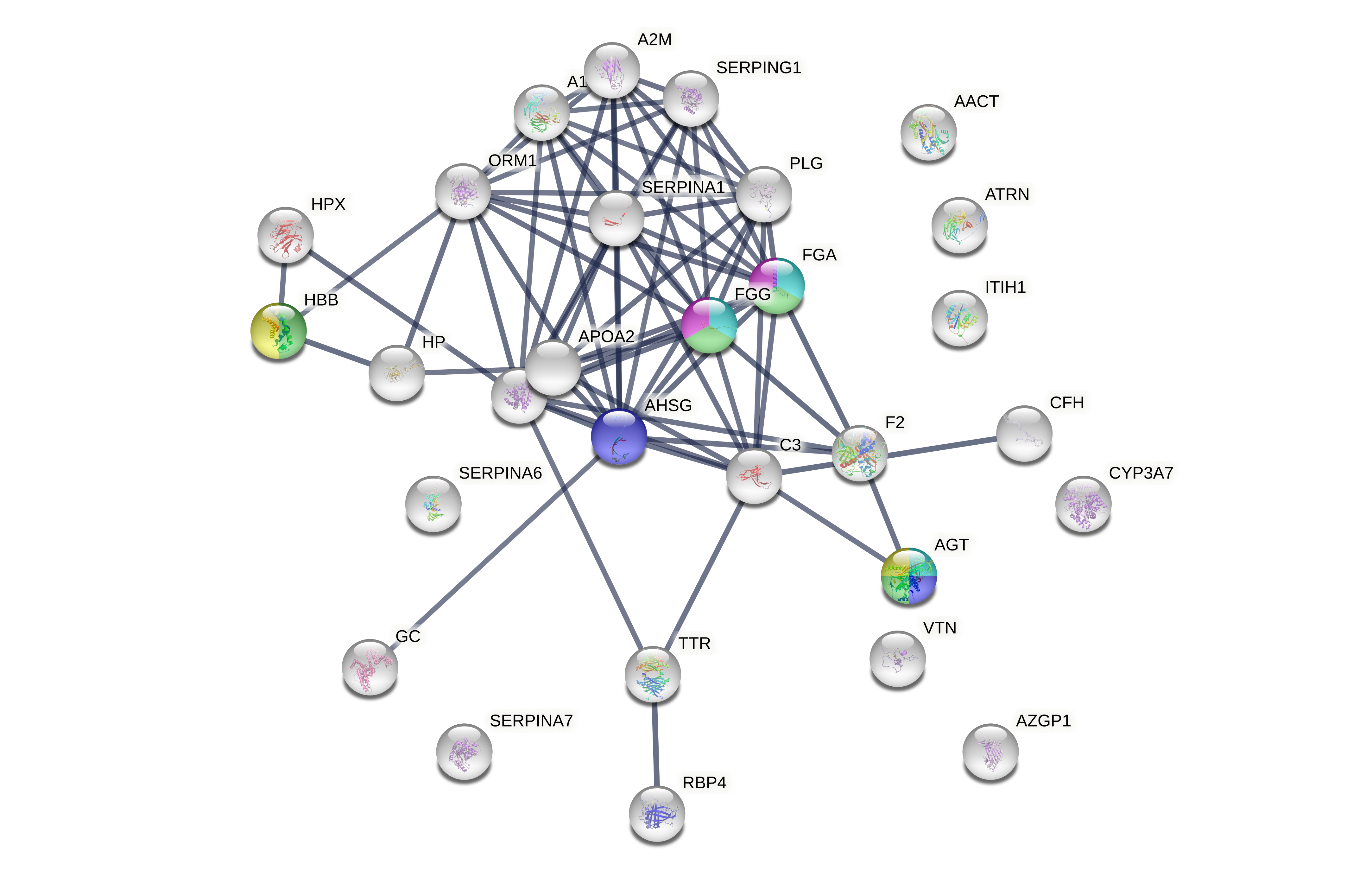

Supplement: Supplementary file 6 — Supplementary Information 6. [file 41598_2021_98672_MOESM6_ESM.png]

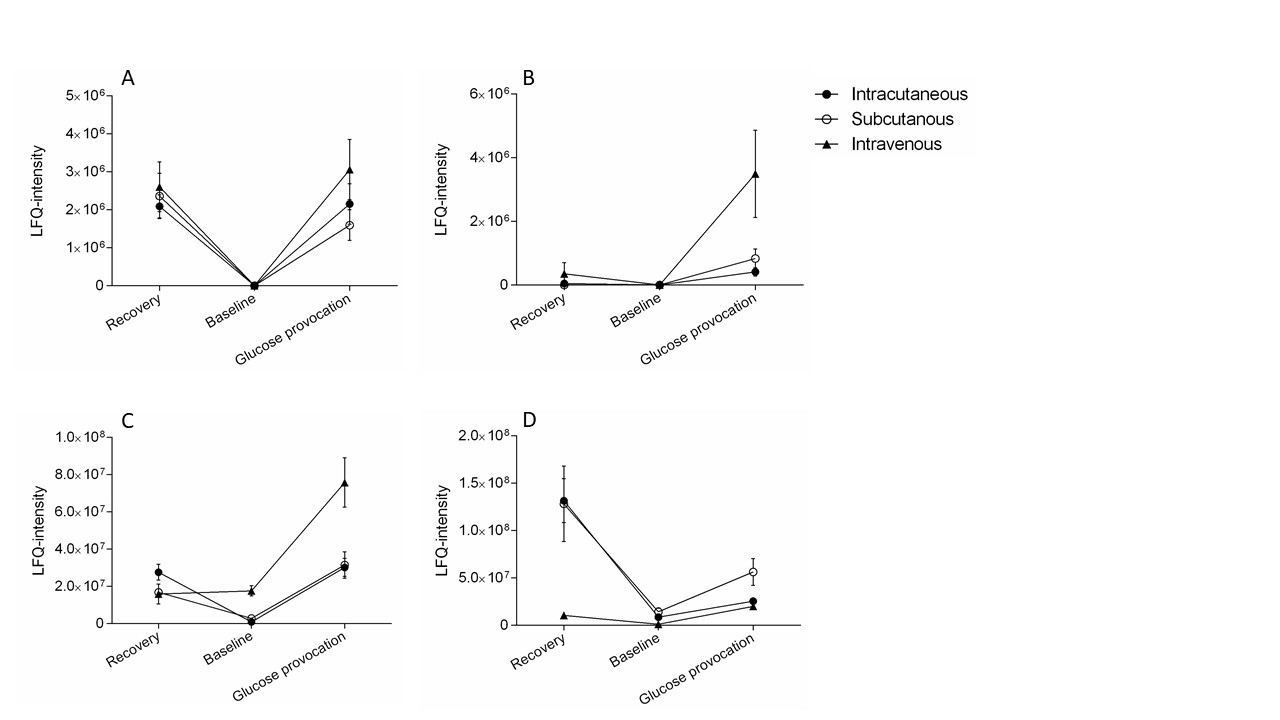

Supplement: Supplementary file 7 — Supplementary Information 7. [file 41598_2021_98672_MOESM7_ESM.jpg]

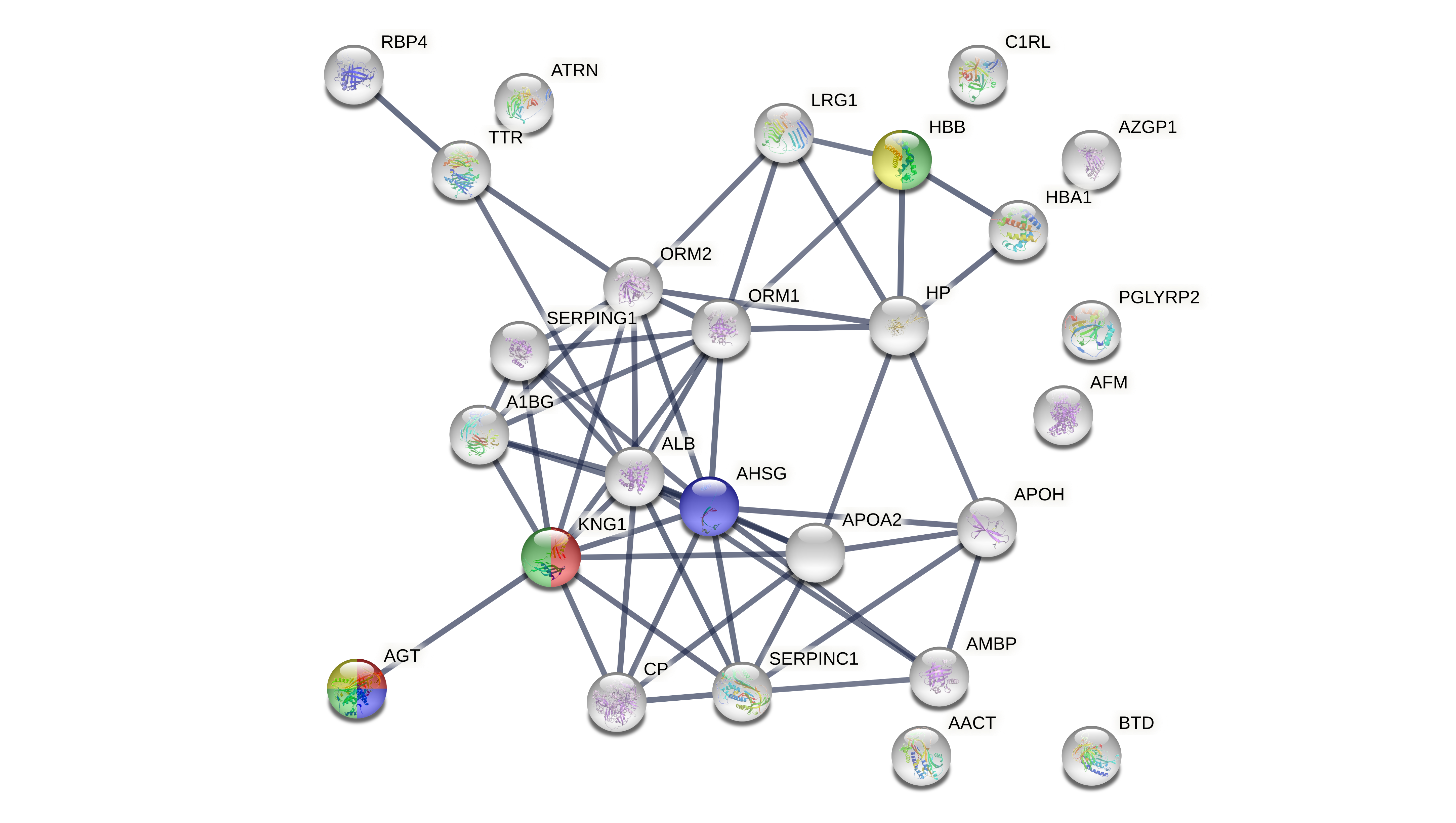

Supplement: Supplementary file 8 — Supplementary Information 8. [file 41598_2021_98672_MOESM8_ESM.png]
