# Supplementary material for: Investigation of proteins important for microcirculation using in vivo microdialysis after glucose provocation: a proteomic study
Source: Sci Rep. 2021 Sep 27;11:19093. doi: 10.1038/s41598-021-98672-8 (PMC8476624; doi:10.1038/s41598-021-98672-8)
Supplement: Supplementary file 9 — Supplementary Legends. [file 41598_2021_98672_MOESM9_ESM.docx]

**Figure legends to supplementary files**

**Figure S1.** Orthogonal partial least square discriminant analysis (OPLS-DA) of the intracutaneous proteome. Score plot showing the differences in intracutaneous protein expression between the different time periods; recovery period after catheter insertion (green), baseline (blue), and glucose provocation (red). R^2 =^ 0.86, Q^2 =^ 0.80, CV-ANOVA p value = 1.70E^-11^. The x-axis represents intergroup differences while the Y-axis represents intragroup differences.

**Figure S2.** Orthogonal partial least square discriminant analysis (OPLS-DA) of the subcutaneous proteome. Score plot showing the differences in subcutaneous protein expression between the different time periods; recovery period after catheter insertion (green), baseline (blue), and glucose provocation (red). R^2 =^ 0.87, Q^2 =^ 0.68, CV-ANOVA p value = 0.3E^-3^. The x-axis represents intergroup differences while the Y-axis represents intragroup differences.

**Figure S3.** Orthogonal partial least square discriminant analysis (OPLS-DA) of the intravenous proteome. Score plot showing the differences in intravenous protein expression between the different time periods; recovery period after catheter insertion (green), baseline (blue), and glucose provocation (red). R^2 =^ 0.77, Q^2 =^ 0.55, CV-ANOVA p value = 0.077. The x-axis represents intergroup differences while the Y-axis represents intragroup differences.

**Figure S4.** Intracutaneous. Pathway analysis of proteins associated with vascular actions of the intracutaneous proteins most important for the separation between the time periods using the STRING database.

Vascular pathways highlighted: red = vasodilation, blue = regulation of cellular response to insulin stimulus, green = regulation of blood vessel diameter, yellow = positive regulation of nitric oxide biosynthetic process. PPI enrichment was < 1.0E^-16^.

Proteins involved in vascular pathways: AGT = Angiotensinogen, KNG = Kininogen-1, AHSG = Alpha-2-HS-glycoprotein, HBB = Hemoglobin subunit beta.

**Figure S5.** Subcutaneous. Pathway analysis of proteins associated with vascular actions of the subcutaneous proteins most important for the separation between the time periods using the STRING database.

Vascular pathways highlighted: red = vasodilation, blue = regulation of cellular response to insulin stimulus, green = regulation of blood vessel diameter, yellow = positive regulation of nitric oxide biosynthetic process. PPI enrichment was < 1.0E^-16^.

Proteins involved in vascular pathways: AGT = Angiotensinogen, KNG = Kininogen-1, AHSG = Alpha-2-HS-glycoprotein, HBB = Hemoglobin subunit beta.

**Figure S6.** Intravenous. Pathway analysis of proteins associated with vascular actions of the intravenous proteins most important for the separation between the time periods using the STRING database.

Vascular pathways highlighted: blue = regulation of cellular response to insulin stimulus, green = regulation of blood vessel diameter, yellow = positive regulation of nitric oxide biosynthetic process, turquoise = regulation of vasoconstriction, purple = positive regulation of vasoconstriction. PPI enrichment was < 1.0E^-16^.

Proteins involved in vascular pathways: AGT = Angiotensinogen, AHSG = Alpha-2-HS-glycoprotein, HBB = Hemoglobin subunit beta, FGA = Fibrinogen alpha chain, FGG = Fibrinogen gamma chain.

**Figure S7.** LFQ-intensity for the proteins involved in vascular actions during the different time periods. A) Angiotensinogen (AGT), B) Kininogen-1 (KNG1), C) Alpha-2-HS-glycoprotein (AHSG), D) Hemoglobin subunit beta (HBB).

**Figure S8.** Pathway analysis of proteins associated with vascular actions of the proteins most important for the separation before (baseline) and after glucose provocation using the STRING database.

Vascular pathways highlighted: red = vasodilation, blue = regulation of cellular response to insulin stimulus, green = regulation of blood vessel diameter, yellow = positive regulation of nitric oxide biosynthetic process. PPI enrichment was < 1.0E-16.

Proteins involved in vascular pathways: AGT = Angiotensinogen, KNG = Kininogen-1, AHSG = Alpha-2-HS-glycoprotein, HBB = Hemoglobin subunit beta.

**Table legends to supplementary files**

**Table S1.** List of all detected proteins in each tissue compartment. Protein ID (accession number) and gene name are referred to according to the protein database Uniprot.

**Table S2.** Significant proteins (VIP >1) in the orthogonal partial least square discriminant analysis (OPLS-DA) model of all three compartments (intracutaneous, subcutaneous and intravenous) that contributed most to the separation between the different time periods, with LFQ-intensity. Data presented in median (min-max). Proteins were considered significant if VIP (variable influence of projection) value was >1. Protein ID (accession number) and gene name are referred to according to the protein database Uniprot (<http://uniprot.org>).

**Table S3.** Significant proteins (VIP >1) in the orthogonal partial least square discriminant analysis (OPLS-DA) model of all three compartments (intracutaneous, subcutaneous and intravenous) that contributed most to the separation in protein expression before (baseline) and after glucose provocation (Figure 4), with LFQ-intensity. Data presented in median (min-max). ↑ = Upregulated; ↓ = Downregulated after glucose provocation compared to before (baseline). Proteins were considered significant if VIP (variable influence of projection) value was >1. Protein ID (accession number) and gene name are referred to according to the protein database Uniprot (<http://uniprot.org>).
